# A Plea for Rationalism in Modern Methods of Practice

**Published:** 1909-07-15

**Authors:** C. N. Johnson

**Affiliations:** Chicago, Ills.


### A PLEA FOR RATIONALISM IN MODERN METHODS OF PRACTICE.

BY C. N. JOHNSON, M.A., L.D.S., D.D.S., CHICAGO, ILLS.

In the rapid evolution of ideas in our profession it behooves us to pause occasionally and consider soberly the real status of some of the new things, and see if in our enthusiasm for the innovations we are not overlooking some of the virtues of older and longer-tried methods. Enthusiasm is a wondrous factor in our professional development. It is the lever which lifts us out of the rut of the commonplace, which stimulates us to shake off the robe of ultra-conservatism, and places us in the van of the glorious progress of the present. Without enthusiasm we should degenerate into dullards, and lose all the inspiration there is in life. Enthusiasm therefore should be cultivated and encouraged in our professional ranks, but it must not be permitted to carry us beyond the bounds of sane and sober reason. Enthusiasm run riot will do more harm than good; it is a marvelous force for advancement but may become dangerous unless controlled by the balance-wheel of judgment.

In a calling like dentistry, where so much of the technical enters into the daily work, it is not strange that one of the glories of the profession relates to the ingenuity displayed by men in the development of new ideas and new methods. It would be a monotonous profession indeed were it not for the possibilities presented by the ever-increasing facilities for performing our operations. But the trouble with some of this ingenuity is that it so frequently leads men to the abandonment of methods which have been so long tested by the profession as to be entitled to be called standard. Nothing is sacred in the old to men who become infatuated with the new. This might be justifiable were it always possible to judge accurately of the ultimate value of the new, but this is not within the pale of human intelligence. The man has not yet been born who can predict with certainty what the future of any method of practice will be. It is true that a passing fad may be so ridiculous that a thinking man can safely prophesy its early abandonment, but the peculiar thing is that this is seldom the kind of prophesying that is done. When the fever of enthusiasm is upon us, we can see nothing but the sunlight and the roses.

The very best of men are subject to mistakes in judgment. At the World's Columbian Dental Congress in 1893, a paper was assigned to the open meeting on the subject of hypnotism. I was on the program committee, and ventured to suggest to those who were in authority in the assignment of papers that it was hardly appropriate that a subject of this character should receive the distinction of presentation before the general body. One of the foremost men in the profession assured me that the essay committee knew precisely what they were doing, and that hypnotism would be an established and universal practice in dentistry in five years from that date.

When cataphoresis was first introduced, I consulted a man who had employed it and questioned him with reference to its virtue. He was a man who is usually extremely conservative, and I had great confidence in his judgment. He said that it was his firm conviction that within five years there would not be a dental office anywhere without a cataphoric outfit, and that it would prove the greatest factor in successful practice that had ever been introduced into dentistry. Five years seems to be the magic time when all these great reforms are to mature, but it has frequently happened in the past that five years has seen the decline, the death, and the burial in oblivion of many of these promising innovations.

When we read and study the history of the past it makes us pause and wonder what may be the future of some of the things we are so enthusiastic about today. Let us strive for mental balance in our progress, and then that progress will be more secure. In the introduction of new methods an unfortunate tendency is the habit of disparaging the old to bolster the new. We should never forget the fundamental fact that to begin by tearing down the old is not always the safest foundation upon which to build the new. In view of the fact that there is so much room in the practice of dentistry for the employment of every good method, it becomes unnecessary to belittle and criticize those things which have been so long in use as to become established practice, just for the purpose of forcing the attention on something recent.

Of all the older methods of practice there is none which has so persistently been the object of disparagement as the process of filling teeth with gold. At repeated intervals thoughout the history of the profession we find men springing up with ever-recurring vigor in a fresh attack upon this method. Every new material that has been introduced into the profession for filling teeth has been heralded as the superior of gold. It is always with gold that a comparison is made, and always to its disadvantage. And yet gold has stood the test of time as has no other material yet introduced. It has been the sheet anchor in saving teeth, while many of its would-be substitutes have fallen by the wayside in the survival of the fittest. This is said at this time not so much with the object of extolling the virtues of gold as to show the folly of criticizing a method which has been so long established.

When the porcelain wave was sweeping over the profession, it was freely prophesied that the day of the foil filling was past, and men in their enthusiasm predicted the relegation of gold pluggers to the junk heap. If we are to believe the reports of manufacturers, to-day porcelain inlay furnaces have become more of a drug on the market than foil pluggers, and yet I wish to go on record as saying that I believe the porcelain inlay has a definite and a very important field of usefulness in our practice. If too much has not been claimed for it in the early days of its introduction, it would have sought its proper level sooner and would have to-day a more firmly established status.

The latest expression of enthusiasm has emanated through the medium of the cast gold inlay. Nothing in recent years has taken so firm a hold on the fancy of the profession as this, and be it said that at present this method bids fairer promise to receive a permanent place in dentistry than has anything that has been introduced in the past decade. But at the risk of being pounced upon by those who are worshipping the new god with so much acclaim, I must affirm that the real and permanent value of the cast gold inlay has not yet been established. Nor can it be, by the very nature of things, till a greater number of years shall have passed than have elapsed since its introduction. It requires time to establish the fact of permanence, and while we have had a sufficiently long experience in its use to prove its title to a firm and definite place in our practice, we do not yet know how long the average cast gold inlay will last. We may say with certainty that it will last long enough to entitle it to a prominent place among our methods, but when we say that it will result in displacing gold pluggers, we are going too far in our predictions unless we wish to acknowledge the ultimate deterioration of dental service. By this I mean that there are many cavities which can be more certainly, more expeditiously, and with better judgment filled with foil than with an inlay. I say more certainly, because I wish at this time to repeat a statement which I have previously made, namely, that there has never yet been devised by the ingenuity of man a filling material which for absolute protection to the tooth, for permanence of service, and for general utility can compare with a perfectly inserted gold foil filling, or, to be more accurate, in selected cases—a platinum-and-gold filling.

It will at once be said in answer to this that the exactions of gold are such that it is well-nigh impossible to insert a perfect foil filling. This is true in certain locations, under certain circumstances, and with certain patients; but it is by no means true of a large percentage of cases which to-day are being filled by some other method. The gold inlay came as a real benefaction to aid us in the management of those cases where the conditions are such that we cannot use gold foil with good technique, but it is being used in many cases where gold foil would insure an infinitely better service.

No one realizes more fully than I the limitations of gold, or its exactions. I have a vivid recollection of the days of long operations and nerve-racking strain, and no human operator would want to go back to that kind of practice. But the improvement in the technique of the gold foil filling renders its insertion a matter of much less discomfort than it formerly used to be, and there should be no excuse on this score for the adoption of some other method in cavities of medium or small size, where good access may be had for perfect adaptation and condensation of the foil. We should not lose sight of the saving properties of gold when inserted under favorable conditions, because it has proved itself to be the most reliable of all materials by years of service, and we should confine our inlay work to cases where the conditions are unfavorable to the proper insertion of gold. Considering it from this point of view, inlay work has come to us as a great and worthy adjunct to our methods of filling teeth, but not to displace older and well-established methods.

Extremes of any kind are proverbially dangerous, and in the wondrous development of new methods, with their fascinating innovations, it seems natural for men to go to extremes. But it is well to call a halt at times and look back over the experience of the past, and see what it teaches us. If this is done calmly and without bias, we shall learn many a useful lesson of conservatism. One of the most significant lessons we shall learn is this—that the chief stumbling block in the way of real progress and the greatest menace to safe practice is the ever constant tendency among the rank and file of the profession to find an easy way of doing things. The search after the short cut has led many a man astray. It is the siren song which has frequently lured the dental mariner to the rocks of failure and defeat. No thinking man will complain of improvements in technique which facilitate our work and enable us to attain our end with less detail, provided the end attained is up to the proper standard of excellence. But too often the short cut results in imperfect work, and consequently in indifferent service to the public.

We hear the constant cry that the practice of dentistry is very exacting, that men break down under the strain, and this is often offered as an excuse for doing things in an easy way. But men break down in every calling in life, and I am not sure that dentists suffer in this regard more than most men. But even if they did, this would be no excuse for adopting methods which are not for the best interests of the public. This one fact should be constantly remembered, that when a man selects a profession as his life-work, he tacitly obligates himself, or at least he should obligate himself, to consecrate his very best energies to the service of humanity, even if this must be done at some self-sacrifice. I am not arguing for a hard way of doing things when there is an easier way which will bring as good results, but I do protest against the constant shirking of the best service simply because that service is hard. In life we develop through difficulties, and professional work is no exception. The greatest happiness is to be attained only through service and self-sacrifice, and it is the chief glory of professional life that it stands apart in this one particular from other callings. It has to do with persons instead of things, and the obligation in respect to service is therefore greater.

What then shall we say of the man that calls himsef a dentist, but who forces upon his unsuspecting patient service of an inferior quality, just because it is easier for him to perform it? This is said not with particular reference to fillings or inlays but as a general protest against the too frequent tendency of adopting a new method and pushing it to an extreme, before it has been fully tried out, just because it possesses the appealing property of being easily performed.

What we most need in the profession is balance, and it would seem as if the experience of the past should teach us a long-needed lesson of conservatism. In nearly every new method that has been introduced we have gone to extremes, frequently to the detriment of the people whom we are supposed to serve. This is the really serious aspect of our over-enthusiasm, that the ones to suffer most are those who repose confidence in us and are thereby entitled to the protection which a wise conservatism on our part should insure them. Take bridge work for instance, and try to estimate the crimes that have been committed in its name, when everything was being bridged from a wabbling third molar to the operator's conscience. Think of the wrecks along the line following the distortion of a system which is really a benefaction when employed with discrimination. Consider cataphoresis, and try to estimate the size of the junk heap that would have reared its lofty head to heaven if all the discarded apparatus had been piled in one place during a single twelve-month. Think of the allurements of the allporcelain plate, the pink celluloid plate, or the filling of glass that could be constructed in a few minutes. Mention has already been made of the rapidly receding wave of porcelain inlay work, and if we were to enumerate all the passing fads that have gripped the profession and have held it in bondage for a period, only to be dropped by the wayside and forgotten it would require a large volume.

These observations should make us pause and consider more calmly the probable outcome of any new idea that is presented, and above all we should not allow the experience of the past to be ruthlessly trampled under foot by the too rapid marching of the present. While we should deem it necessary in the evolution of the pofession to prove all things new, it is just as incumbent upon us to hold fast to that which is good in the old.

This is a most inspiring day in dentistry, and we may well be pardoned for all joining in a grand chorus of acclaim over the wondrous advances of the present, but in doing this let us not forget to pause occasionally and peal a swelling anthem of praise to the glorious heritage of the past.—June *Cosmos.*

